# Disseminated histoplasmosis in a paediatric patient at a referral hospital in Nairobi, Kenya

**DOI:** 10.11604/pamj.2019.34.139.20685

**Published:** 2019-11-08

**Authors:** Anne-Marie Macharia, Edwin Oloo Walong

**Affiliations:** 1 Unit of Clinical Infectious Disease, Department of Clinical Medicine and Therapeutics, University of Nairobi, Nairobi, Kenya; 2 Department of Human Pathology, University of Nairobi, Nairobi, Kenya

**Keywords:** Paediatric, histoplasmosis, disseminated

## Image in medicine

A two year old boy presented with two-month history of fever and abdominal swelling. He developed jaundice and had experienced significant weight loss. There was no history of tuberculosis contact. At presentation the patient was febrile with tachypnoea and tachycardia. He had pallor, jaundice, generalized lymphadenopathy, petechiae, gingival bleeding and epistaxis. The liver was palpable 8cm below the costal margin with splenomegally 9cm below the costal margin. Laboratory investigations were notable for severe anaemia with leucopenia and severe thrombocytopenia. CRP was elevated at 55mg/l. He was HIV negative. Liver and renal function were normal. The chest radiograph was unremarkable. Abdominal ultrasound showed enlarged homogenous liver, span 9.2cm, with no focal masses. The spleen was enlarged with a span of 10.8cm. There was no adenopathy or ascites. A bone marrow aspirate was done, results below. What is your diagnosis? Treatment was started with amphotericin B deoxycholate, but he succumbed after 5 days of treatment. Bone marrow aspirate revealed macrophages with severe infestation by fungal spores of histoplasma. Histoplamosis is a dimorphic fungus endemic in parts of Africa. Severe and disseminated infection occurs in immunosuppression and at extremes of age. Our patient presented with lymphadenopathy and hepatosplenomegally, suggesting disseminated histoplamosis. Severe cases are treated with amphotericin B which rapidly eradicates fungaemia, then transitioned to itraconazole to complete at least one year of treatment.

**Figure 1:**
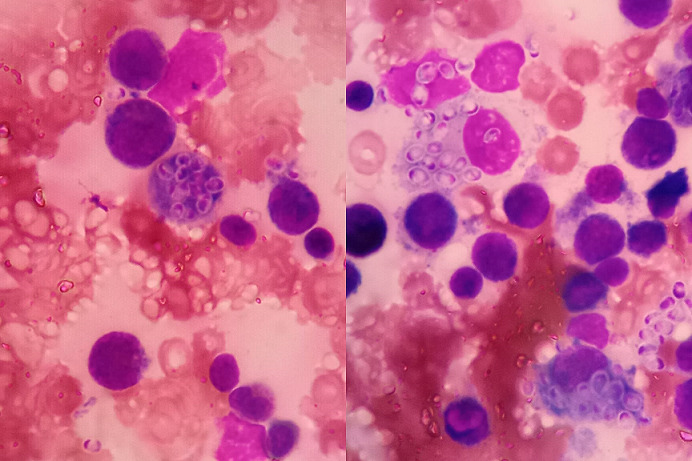
The bone marrow cytology preparation shows macrophages whose cytoplasms have abundant intracellular microorganism, with some present within interstitial spaces. These are 2-4 micrometer yeast cells that have eccentric staining. Characteristic narrow budding is seen in some cysts. Morphologically, these are consistent with *Histoplasma capsulatum*

